# Plant‐Trait Syndromes and Environmental Filtering Drive Biomass Ecology in Resource‐Limited Forest Ecosystems

**DOI:** 10.1002/ece3.73949

**Published:** 2026-07-07

**Authors:** Shahab Ali, Shujaul Mulk Khan, Henrik Balslev, David P. Edwards

**Affiliations:** ^1^ Department of Plant Sciences and Centre for Global Wood Security University of Cambridge Cambridge UK; ^2^ Department of Plant Sciences Quaid‐i‐Azam University Islamabad Pakistan; ^3^ Department of Biology ‐ Ecoinformatics and Biodiversity Aarhus University Denmark; ^4^ Pakistan Academy of Sciences Islamabad Pakistan; ^5^ Conservation Research Institute University of Cambridge Cambridge UK

**Keywords:** climate, deciduous, Evergreen, functional diversity, functional dominance, mixed, soil

## Abstract

Understanding the mechanisms driving aboveground biomass (AGB) variation in forest ecosystems is essential for biodiversity conservation and climate‐change mitigation, particularly in environmentally heterogeneous and resource‐limited regions. Drawing on community assembly theory and trait‐based ecology, this study examines how functional dominance and functional diversity interact with soil fertility and climate to regulate AGB across deciduous, evergreen, and mixed forest stands in Pakistan. Using a combination of generalized linear modeling and machine‐learning approaches, we found that AGB is primarily structured by functional dominance, with soil fertility exerting an additional but secondary influence. Climatic factors generally constrained biomass accumulation, although their importance varied among forest stand types. Functional diversity played a comparatively minor role. Forest stand type further modulated these relationships, with mixed forests supporting higher biomass and exhibiting the strongest influence of dominant functional traits, while climate effects were more pronounced in evergreen forests and negligible in mixed stands. Overall, our findings indicate that in resource‐limited forest ecosystems, biomass accumulation is governed more by the dominance of particular functional strategies and soil resource availability than by functional diversity or climate alone. Integrating plant functional traits and soil conditions into forest management and restoration strategies may therefore enhance biomass productivity and carbon sequestration in heterogeneous landscapes.

## Graphical abstract

Aboveground biomass (AGB) in Pakistan's forests is primarily driven by functional dominance, with soil fertility playing a secondary but significant role, while climate effects vary across forest types. Mixed forests support the highest biomass and show the strongest influence of dominant traits, whereas climate constraints are more evident in evergreen stands. These findings highlight that biomass accumulation in resource‐limited ecosystems is shaped more by dominant functional strategies and soil resources than by functional diversity alone.

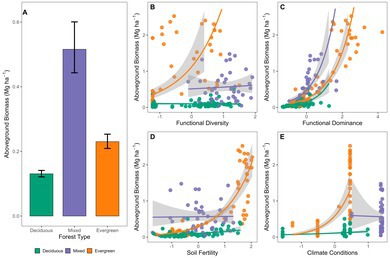

## 1 Introduction

Forests are essential ecosystems that play a central role in global carbon dynamics, biodiversity conservation, and ecosystem service provision (Pan et al. 2011; Tian et al. 2023; Zhang et al. 2025). Aboveground biomass (AGB), representing the carbon stored in living vegetation, is a widely used indicator of forest productivity and carbon sequestration potential because it integrates long‐term growth, survival, and carbon allocation strategies of forest communities (Chave et al. 2014; Ma et al. 2024). Understanding the ecological drivers of AGB is crucial for predicting forest responses to environmental change and for designing sustainable management strategies (Pan et al. 2013; Poorter et al. 2015). Global studies have revealed how plant trait diversity and identity shape AGB (Wang, Hou, et al. 2023; Wang, Song, et al. 2023), with morphological, physiological, and phenological attributes influencing plant performance through effects on growth rates, tissue longevity, wood density, and competitive ability, making them powerful predictors of ecosystem function (Díaz et al. 2007; Mensah et al. 2024; Li and Prentice 2024).

Although traits like wood density can enhance biomass (Prado‐Junior et al. 2016), the influence of functional traits varies across environments (Fortunel et al. 2014; Li and Prentice 2024). For example, in the Amazon Basin, species with high wood density tend to dominate in nutrient‐poor upland forests, where structural strength and longevity are advantageous, whereas in seasonally flooded varzea forests, species with lower wood density and rapid growth rates contribute more substantially to biomass accumulation (Ter Steege et al. 2006; Poorter et al. 2010). Similarly, in Central African tropical forests, high wood density species prevail in drier, more seasonal regions, enhancing resistance to drought‐induced mortality, while in wetter, less seasonal areas, fast‐growing, low wood density species achieve higher biomass turnover rates (Fayolle et al. 2012; Lewis et al. 2013). Recent studies further demonstrate that the effects of functional traits on biomass and carbon storage are strongly mediated by climatic and edaphic conditions, emphasizing the context dependency of trait–ecosystem functioning relationships across forest biomes (Li and Prentice 2024; Mensah et al. 2024). These metrics represent distinct ecological mechanisms that may contribute differently to AGB depending on environmental conditions.

Trait‐based ecology emphasizes species traits over taxonomic identity, offering insights into community assembly across environmental gradients (McGill et al. 2006). Two main theoretical frameworks guide this approach: the mass‐ratio hypothesis, where dominant species' traits determine ecosystem functions (Grime 1998); and the niche complementarity hypothesis, which posits that trait diversity enhances productivity via resource partitioning (Tilman et al. 1997; Loreau and Hector 2001). Community assembly theory explains biomass–trait relationships through mechanisms such as environmental filtering, niche differentiation, and limiting similarity (MacArthur 1969; Westoby et al. 2002). Functional traits act as measurable proxies for these processes (Díaz et al. 2004; Reich 2014), linking species characteristics to landscape‐scale biomass patterns (Laliberté and Legendre 2010; Kunstler et al. 2016). Functional diversity enhances ecosystem multifunctionality and resilience, with biodiversity buffering against climatic and anthropogenic disturbances (Mouillot et al. 2013; Díaz et al. 2016; Isbell et al. 2023). Environmental filtering, where climate and soil variables limit species presence and function, is a key driver in shaping trait distributions and forest structure (Keddy 1992; Kraft et al. 2015). Climate factors (e.g., temperature, precipitation, humidity) regulate photosynthesis and growth (Huang et al. 2017), while soil characteristics (e.g., nutrient content, texture, pH) affect resource availability (Wright et al. 2004; Chen et al. 2023). Through long‐term environmental filtering, these abiotic factors give rise to distinct forest stand types—deciduous, evergreen, and mixed—which represent characteristic assemblages of functional traits rather than independent mechanistic drivers of biomass (Chazdon et al. 2007; Mascaro et al. 2011).

Two core metrics can be used to quantify trait influence on AGB. First, functional dominance is represented by community‐weighted means (CWM) of plant traits—including maximum tree height (Hmax), maximum diameter at breast height (DBHmax), maximum crown area, specific leaf area (SLA), and leaf dry matter content (LDMC). These traits reflect the ecological strategies of dominant species that disproportionately influence biomass accumulation (Garnier et al. 2004; Lavorel et al. 2008; Li and Prentice 2024; Bruelheide et al. 2018). Structural traits determine canopy occupation and resource capture, whereas leaf economic traits reflect acquisitive–conservative resource‐use strategies that influence growth and carbon gain (Conti and Díaz 2013; Mensah et al. 2024; van der Sande et al. 2017). The relative importance of functional dominance and functional diversity varies across forest types and successional stages. Early‐successional deciduous stands are typically characterized by acquisitive trait syndromes, including high specific leaf area, rapid growth rates, and efficient resource capture, facilitating rapid biomass accumulation (Reich et al. 1997; Wright et al. 2004; Poorter et al. 2008). Mid‐successional stands often support the coexistence of deciduous and evergreen species, increasing trait heterogeneity and niche complementarity, thereby enhancing resource‐use efficiency and ecosystem functioning (Tilman et al. 1997; Cadotte 2011; Díaz et al. 2016). Late‐successional evergreen stands are generally dominated by conservative trait strategies, such as high leaf dry matter content, greater tissue longevity, and enhanced stress tolerance, which promote persistence under resource‐limited conditions (Reich 2014; Díaz et al. 2004; Poorter et al. 2015). Climate and soil fertility influence the rate and direction of successional shifts by acting as environmental filters favoring either acquisitive or conservative trait syndromes (Keddy 1992; Cornwell and Ackerly 2009; Jiang et al. 2023). Consequently, productive environments support greater functional complementarity, whereas nutrient‐poor soils and climatic stress favor functional dominance by conservative species adapted for persistence (Jucker et al. 2018; Bastin et al. 2017; Li and Prentice 2024). These dynamics influence biomass accumulation and variation in aboveground biomass among forest stands (Conti and Díaz 2013; Bruelheide et al. 2018; Wang, Hou, et al. 2023; Wang, Song, et al. 2023). This framework aligns with the mass‐ratio hypothesis, which proposes that ecosystem properties are primarily driven by the traits of the dominant species rather than by species richness alone (Conti and Díaz 2013; Wang, Hou, et al. 2023; Wang, Song, et al. 2023; Isbell et al. 2023).

Second, functional diversity describes the extent of trait variation among species and is commonly measured using indices such as functional richness, evenness, divergence, and dispersion. These indices capture niche differentiation and trait complementarity among coexisting species (Díaz et al. 2016; Carmona et al. 2016; Rosenfield et al. 2023). Higher functional diversity may enhance AGB through complementary use of resources (Tilman et al. 1997; Flynn et al. 2011). However, these effects are often weak or context‐dependent, particularly under strong environmental filtering (Finegan et al. 2015; Jucker et al. 2018).

While trait–AGB relationships have been extensively studied in productive tropical forests, they remain less understood in resource‐limited forest systems, including seasonally dry tropical forests, subtropical mountain forests, semi‐arid woodlands, and forests on shallow or nutrient‐poor soils (Mensah et al. 2021; Dexter et al. 2018; Jucker et al. 2018). In these ecosystems, limited water and nutrient availability constrain productivity and biomass accumulation. Strong abiotic filters favor conservative functional strategies and may alter the relative importance of functional dominance and diversity in regulating AGB (Bastin et al. 2017; Yanhui et al. 2026). According to environmental filtering theory, stressful conditions promote trait convergence toward conservative strategies that enhance persistence under harsh conditions (Keddy 1992; Jiang et al. 2023). These trait syndromes often support biomass stability rather than rapid growth, resulting in AGB dynamics distinct from those of highly productive forests (Díaz et al. 2004; Grime 2006; Li and Prentice 2024). Therefore, understanding how functional dominance and diversity regulate AGB is essential for sustainable forest management and effective restoration in resource‐limited regions (Ali et al. 2022; Mensah et al. 2024).

In this study, we address the core ecological question of what regulates aboveground biomass by testing a conceptual framework in which AGB is influenced by functional dominance and functional diversity of plant traits, under the combined effects of climate conditions and soil fertility as key environmental factors, with resource‐limited forest types providing an ecological context rather than acting as causal processes themselves. This integrative framework is novel in simultaneously linking functional trait structure, stand composition, and multi‐source environmental filtering to explain spatial variation in AGB. We hypothesize that the relative importance of functional dominance and functional diversity in determining AGB differs among deciduous, evergreen, and mixed forest stands, reflecting variation in environmental filtering strength and resource limitation. We further expect functional dominance to play a stronger role in more environmentally constrained forest types, whereas functional diversity may contribute more under relatively less restrictive conditions. We focus on Pakistan, a country that encompasses a wide range of forest ecosystems. The selected forest stands—deciduous, mixed, and evergreen—represent a clear successional and functional gradient in dominant vegetation types, ranging from fast‐growing, disturbance‐adapted communities to structurally complex, late‐successional coniferous forests. This gradient is particularly suitable for testing trait‐based controls on biomass because it captures systematic variation in resource availability, disturbance history, and environmental filtering across a relatively compact geographic region. (Ali, Khan, Ahmad, Abdullah, et al. 2023). This ecological diversity, combined with increasing anthropogenic pressures, makes Pakistan an ideal natural laboratory to investigate how biodiversity, structure, and environmental filters shape aboveground biomass. Although the study is grounded in Pakistan, the proposed framework and findings are relevant to broader dry temperate and subtropical forest ecosystems globally, where similar interactions between climate, soil fertility, and functional traits regulate forest carbon storage.

## 2 Methods

### 2.1 Study Area

**FIGURE 1:**
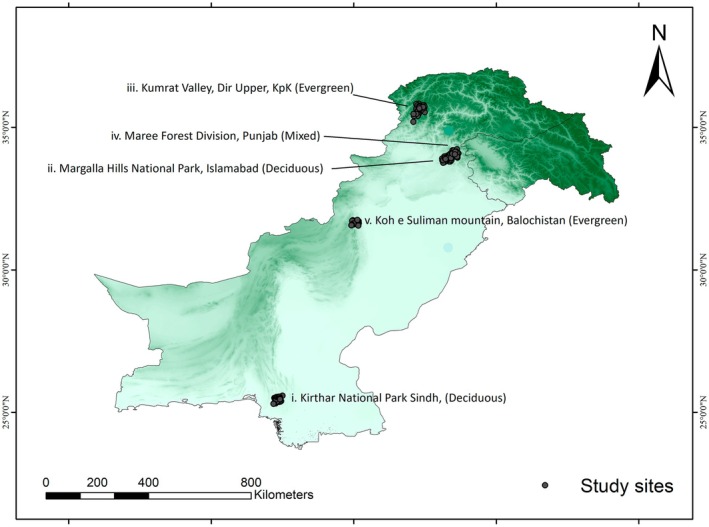
Location of the forests studied in Pakistan.

Pakistan is endowed with a remarkably diverse geography, climate, and forest cover, with elevations ranging from sea level to 8611 m. This variation supports a wide range of forest types, including Dry Subtropical Forests, Moist Subtropical Broad‐leaved Forests, Moist Temperate Mixed Forests, Dry Temperate Conifer Forests, and Dry Temperate Pure *Pinus gerardiana* Forests. The country's northern landscape is dominated by three major mountain ranges the Karakoram, Hindu Kush, and the Himalayas which collectively cover about 60% of the northern territory. These ranges feature snow‐capped peaks, deep valleys, and inner mountain basins. Additional prominent ranges include the Suleiman Mountains in Baluchistan and the Kirthar Range in Sindh, both of which contribute significantly to Pakistan's ecological diversity (Ali, Khan, Ahmad, Siddiq, et al. 2023). In this study, we examined three forest stand types Deciduous, Evergreen, and Mixed across five representative forest ecosystems that span the major ecological zones of the country. These selected forests capture the structural and functional variation of Pakistan's dominant vegetation types. The Deciduous forest stands include the subtropical forest of the Kirthar Range in southwestern Sindh, a Category II protected area under the IUCN classification (Enright et al. 2005), and the subtropical broad‐leaved forest of the Margalla Hills National Park in the western Himalayan foothills, covering 126.05 km^2^ (Ali et al. 2022). The Evergreen forest stands consist of dry temperate conifer forests in Dir Upper, where tall, large‐diameter coniferous trees thrive in inner valleys (Hazrat et al. 2011), and the dry temperate pure pine forests of the Shirani Suleiman Range Baluchistan at the junction of Punjab and Khyber Pakhtunkhwa, which include the world's largest natural stand of Chilghoza pine ( *Pinus gerardiana* ), spanning 260 km^2^ between 500 and 3441 m in elevation (Ali, Khan, Ahmad, Abdullah, et al. 2023). The Mixed forest stands are represented by the temperate mixed forests of Murree and Ayubia, situated between 1041 and 2566 m above sea level, composed predominantly of various *Pinus* species interspersed with broad‐leaved trees. Together, these forests encompass the majority of Pakistan's forest vegetation and ecological zones, making them highly representative for investigating the effects of functional traits, environmental factors, and forest structure on aboveground biomass dynamics (Figure 1 and Appendix S1).

### 2.2 Forest Inventory

A total of 200 plots (each measuring 20 × 20 m; total area = 8 ha) were established across different forest stand types between March and September 2020. These included 80 plots (3.2 ha) in deciduous forests, 80 (3.2 ha) in evergreen forests, and 40 (1.6 ha) in mixed forest stands. The plots were separated by a distance of 1 km or a 20‐m difference in elevation. All woody plants in the plots with a diameter at breast height (DBH) < 1 cm were identified by a taxonomist and confirmed at (http://www.theplantlist.org/tpl1.1/search at http://www.efloras.org/flora_page.aspx?flora_id=5). The number of individuals per species was counted for the calculation of functional diversity and functional dominance. The diameter of the tree at the height of the breast was recorded using a measuring tape. Crown area for each tree was calculated using the formula for the area of an ellipse:

(1) $$\left(\mathrm{Ae}\right):\mathrm{Ae}=\pi \;\left(0.5x\right)\times \left(0.5y\right)$$

where (*x*) is the length of the crown and (*y*) is the width measured perpendicularly (Ali et al. 2022; Ali, Khan, Ahmad, Abdullah, et al. 2023). The height of the tree was calculated using a clinometer (Dănescu et al. 2016). To assess the functional traits of each species present in the plots, we sampled approximately 10 cm segments of wood (up to 0.5 cm in diameter) and collected 10–15 leaves, depending on the plant type, from fresh and sun‐exposed branches of three individuals per species. These samples were used to determine specific wood density and specific leaf area (SLA), leaf dry matter content (LDMC) (Wang et al. 2025). Elevations were recorded using a GPS (geographical positioning system). Soil samples were collected from each plot up to a depth of 10 cm and kept in polythene bags for further analysis.

### 2.3 Assessment of Aboveground Biomass

For the aboveground biomass (AGB), we use the following allometric equation developed by Chave et al. (2014).

(2) $$\mathrm{AGB}=0.0673\times {\left(\mathrm{WD}\times {\mathrm{DBH}}^{2}\times H\right)}^{0.976}$$

Here, *WD* represents dry wood density, *DBH is* the diameter of the tree at breast height, and *H is* the height of the tree. This pantropical allometric model was selected because it has been extensively validated across a wide range of tropical and subtropical forest ecosystems and incorporates key tree attributes known to strongly influence biomass estimation. In the absence of species‐specific or regionally calibrated allometric equations for the diverse tree species included in this study, the Chave et al. (2014) model provides a robust and widely accepted approach for estimating aboveground biomass (Basuki et al. 2009; Feldpausch et al. 2012; Vieilledent et al. 2012; Lewis et al. 2013). For gymnosperm species, species‐specific allometric equations were applied to estimate the aboveground biomass (AGB) of individual trees, as recommended by previous studies (Ali, Khan, Ahmad, Abdullah, et al. 2023; Ismail et al. 2018; Ullah et al. 2021). These equations (3–7), derived from regionally appropriate data (Jenkins et al. 2003), incorporate diameter at breast height *DBH* and, where applicable, tree height *H*. The AGB of each tree was calculated using the corresponding species‐specific equation and subsequently summed for all trees within each plot.

The applied equations were as follows:

(3) $$\text{Abies pindrow}:\mathrm{AGB}=0.0954\times {\left({\mathrm{DBH}}^{2}H\right)}^{0.8114}$$

(4) $$\text{Cedrus deodara}:\mathrm{AGB}=0.1779\times {\left({\mathrm{DBH}}^{2}H\right)}^{0.8103}$$

(5) $$\text{Picea smithiana}:\mathrm{AGB}=0.0843\times {\left({\mathrm{DBH}}^{2}H\right)}^{0.8472}$$

(6) $$\text{Pinus wallichiana}:\mathrm{AGB}=0.0631\times {\left({\mathrm{DBH}}^{2}H\right)}^{0.8798}$$

(7) $$\text{Pinus gerardiana}:\mathrm{AGB}=0.0253\times {\mathrm{DBH}}^{2.6077}$$

### 2.4 Assessment of Plant Functional Traits

We used forest inventory data collected across multiple forest types in Pakistan, comprising species‐level abundance and functional trait information. Functional traits were selected based on their established ecological relevance to plant productivity, competitive ability, and carbon storage. To represent resource acquisition strategies and plant size, we quantified specific leaf area (SLA), leaf dry matter content (LDMC), maximum plant height (Hmax), maximum diameter at breast height (DBHmax), and maximum crown area (Crownmax). These traits capture key dimensions of the leaf economics spectrum, tissue construction costs, and competitive ability for light interception, and are widely used in trait‐based ecological studies (Wright et al. 2004; Díaz et al. 2016; Poorter et al. 2015; Bruelheide et al. 2018; Li and Prentice 2024). Trait values were obtained from field measurements. Functional dominance (FDominance) was quantified using the community‐weighted mean (CWM) of each trait at the plot level. CWM reflects the average trait value weighted by species relative abundance and captures the influence of dominant species on ecosystem functioning (Garnier et al. 2004; Lavorel et al. 2008; Conti et al. 2023; Mensah et al. 2024). The CWM for trait *t* in plot *i* was calculated as:

(8) $$\mathrm{CWM}i,t=\sum_{i=j}^{s}\mathrm{pij}.\mathrm{xjt}$$

where *pij* is the relative abundance of species *j* in plot *i*, *xjt* is the value of trait *t* for species *j*, and *S* is the total number of species in plot *i*. CWMs were calculated for SLA, LDMC, DBHmax, Crownmax, and Hmax. To avoid circularity between biomass estimation and trait‐based predictors, wood density was deliberately excluded from the calculation of FDominance, as it was already used in allometric equations to estimate aboveground biomass. Functional diversity (FD) was quantified independently using functional richness (FRic), functional evenness (FEve), functional divergence (FDiv), and functional dispersion (FDis), which collectively describe the distribution of species within a multidimensional trait space and their ecological differentiation. FRic represents the volume of trait space occupied by the community, FEve reflects the evenness of species abundances within that space, FDiv captures the dominance of extreme trait values consistent with niche differentiation, and FDis represents the abundance‐weighted dispersion of traits around the community centroid.

Functional diversity indices were calculated using the *FD* package in R (Laliberté and Legendre 2010). Trait values were standardized (mean = 0, SD = 1), and Gower distance matrices were computed to account for differences in trait scales. Principal Coordinates Analysis (PCoA) was then used to construct the multidimensional functional trait space, and FD indices were calculated separately for each plot to characterize within‐community functional structure.

### 2.5 Assessment of Environmental Factors

To quantify soil nutrient concentrations, specifically Chromium (Cr), Nickel (Ni), Copper (Cu), Manganese (Mn), Calcium (Ca), Magnesium (Mg), Sodium (Na), Cobalt (Co) the acid digestion method was employed for sample preparation following the procedures outlined by Filgueiras et al. (2000) and Zasoski and Burau (1977). One gram of oven‐dried and finely ground soil (in three replicates per plot) was weighed and placed into a 50 mL conical flask. A 10 mL acid mixture, consisting of nitric acid (HNO_3_) and perchloric acid (HClO_4_) in a 3:1 ratio, was added to each sample for pre‐digestion, which proceeded for 24 h at room temperature. Following pre‐digestion, the samples were heated in a fume hood at 150°C for 1 h to initiate digestion. The temperature was then increased gradually to 235°C until the appearance of white fumes, indicating complete digestion. After cooling, the digested mixture was filtered, and the final volume was brought to 40 mL using distilled water (Ahmad et al. 2019). Elemental concentrations of Chromium, Nickel, Copper, Manganese, Calcium, Magnesium, Sodium, and Cobalt in the digested soil samples were analyzed using an Atomic Absorption Spectrophotometer (VARIAN, AA240FS). Results were reported in milligrams per kilogram (mg/kg) of dry soil (Malik et al. 2010). Soil nitrogen content was determined by sending samples to the Rawalpindi Agriculture Center, where analyses followed the protocol of Bremner (1996). Climatic factors such as Mean annual temperature (Mt), mean annual precipitation (MP), mean wind pressure (MWP), and mean relative humidity (MRH) were obtained from the Pakistan Metrological Department Islamabad.

### 2.6 Statistical Analysis

All statistical analyses were conducted in R (version ≥ 4.3.0) using the packages *tidyverse*, *broom*, *emmeans*, *performance*, *relimpo*, *randomForest*, and *ggpubr*, with aboveground biomass (AGB; Mg ha^−1^) used as the response variable. To address potential circularity and non‐independence between functional traits and aboveground biomass (AGB), particularly for traits directly involved in biomass estimation, such as tree height and diameter at breast height (DBH), we implemented a null model approach based on permutation tests applied to the functional trait matrices. This was particularly important to ensure that observed relationships were not artificially inflated due to inherent dependencies, especially for size‐related traits. For each trait, the observed Pearson correlation coefficient (r) between plot‐level community‐weighted mean (CWM) values. A null distribution of correlation coefficients was then generated by randomly permuting trait values across plots (*n* = 999 iterations), while keeping AGB values fixed. This procedure removes any real association between traits and biomass, thereby providing a baseline expectation under random conditions. Statistical significance was assessed using a two‐tailed test, where the *p*‐value was calculated as the proportion of null correlations with absolute values greater than or equal to the observed correlation: Equation used for permutation‐based *p*‐value calculation:

(9) $$p=\frac{\#\left(r\;\text{null}|\geq |\;r\;\mathrm{obs}\right)}{n}$$

where *r_obs* is the observed correlation coefficient, *r_null* represents correlation coefficients from permuted datasets, and *n* is the number of permutations (999). Traits were considered significantly associated with AGB when the observed correlation deviated from the null expectation (*p* < 0.05). Only those traits showing robust and non‐random relationships with AGB were retained as final variables for subsequent analyses.

To reduce multicollinearity and summarize correlated predictors, principal component analysis (PCA) was applied separately to soil, climate, and functional trait datasets. Soil fertility was represented by the first PCA axis derived from nine edaphic variables (i.e., Cr, Ni, Cu, Mn, Ca, Mg, Na Co, and total N), capturing variation in macro‐ and micronutrient availability relevant to plant growth in these forest systems. Climate conditions were summarized using mean temperature (Mt), mean precipitation (MP), mean wind pressure (MWP), and mean relative humidity (MRH), reflecting broad climatic constraints on forest productivity. Functional traits were quantified using two complementary indices reflecting distinct ecological mechanisms. Functional dominance (FDominance) was calculated as the first PCA axis of community‐weighted mean (CWM) traits, including specific leaf area (SLA), leaf dry matter content (LDMC), maximum tree height (Hmax), maximum diameter at breast height (DBHmax), and maximum crown area (Crownmax). These traits capture acquisitive–conservative strategies and size‐related attributes of dominant species, consistent with the mass‐ratio hypothesis. Wood density was explicitly excluded from functional dominance metrics to avoid circularity, as it was used in the allometric equations for estimating AGB. Functional diversity (FD) was summarized as the first PCA axis derived from FRic, FEve, FDiv, and FDis, representing multidimensional trait dispersion and niche complementarity among coexisting species. To quantify the relative contributions of functional traits, environmental variables, and forest stand types to AGB, we employed a hierarchical multi‐model analytical framework. This strategy allows robust inference while avoiding strong a priori assumptions about causal structure. First, Gamma generalized linear models (GLMs) with a log link were used to evaluate the joint effects of functional dominance, functional diversity, soil fertility, climate conditions, and forest stand type on AGB. This approach accommodates the right‐skewed distribution typical of biomass data and allows explicit testing of additive effects across forest types. Estimated marginal means (EMMs) were back‐transformed for interpretation and used to compare AGB among forest types. Second, multiple linear regression (MLR) was applied to assess linear relationships among predictors. The independent contribution of each predictor was quantified using the Lindeman–Merenda–Gold (LMG) method, which partitions explained variance while accounting for correlations among predictors. Third, to capture non‐linear relationships and potential interactions, Random Forest models were fitted separately for each forest type (Deciduous, Evergreen, and Mixed). Variable importance was assessed using the increase in node purity metric and expressed as relative contributions within each forest type. This forest‐type‐specific approach explicitly tests whether the drivers of AGB differ among forest types, thereby addressing ecological heterogeneity without imposing causal assumptions. Furthermore, a full‐variable MLR including all original functional traits, functional indices, soil variables, climate variables, and forest type was fitted to examine individual predictor effects without dimensional reduction, providing an additional robustness check. Finally, descriptive statistics were calculated for species and stand characteristics, including mean values, standard errors, abundance, relative abundance, frequency, relative frequency, and other community composition metrics to characterize vegetation structure and species distribution patterns.

## 3 Results

### 3.1 Effects of Functional Dominance, Functional Diversity, Soil Fertility, Climate, and Forest Type on Aboveground Biomass

**FIGURE 2:**
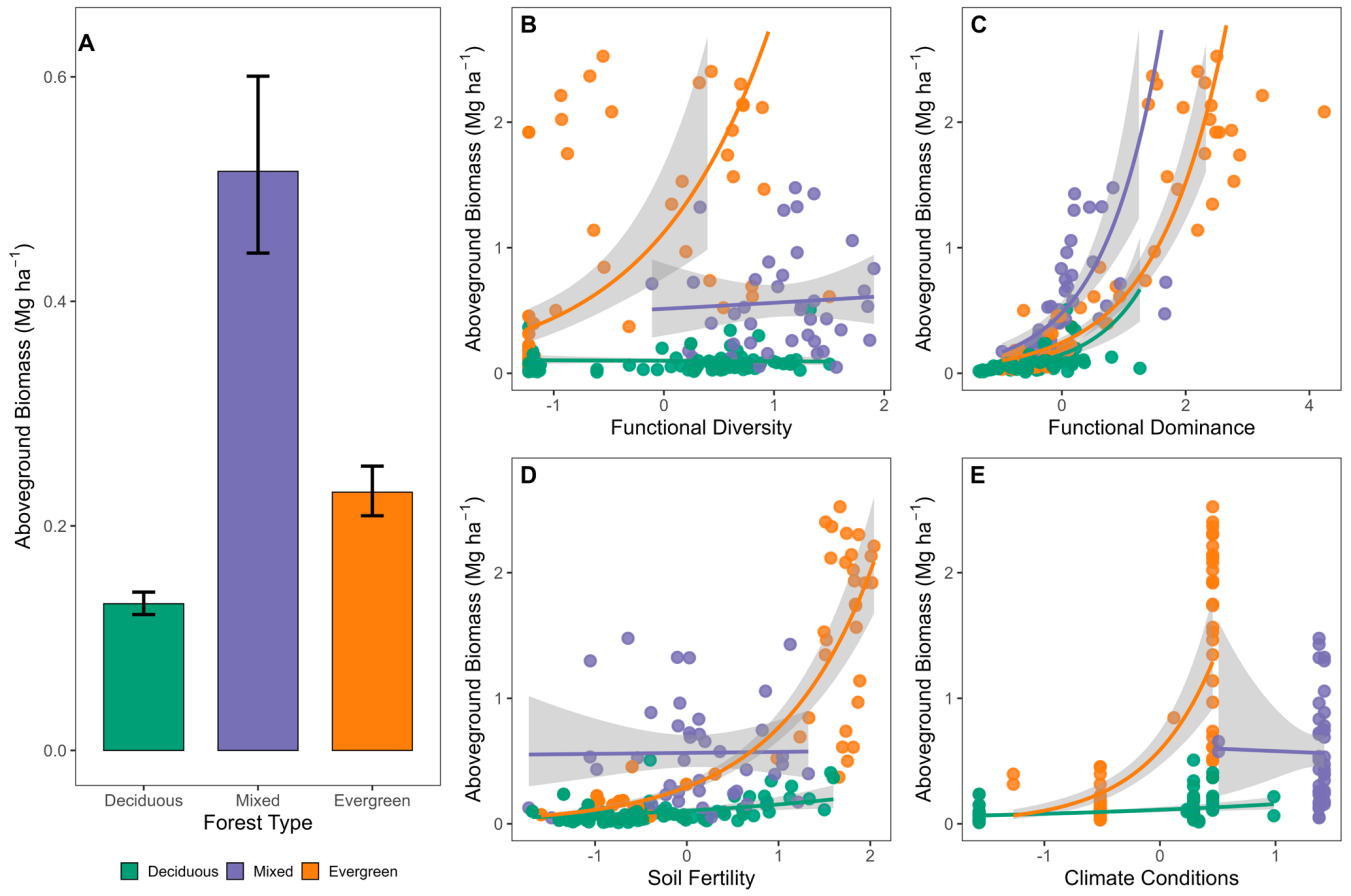
Effects of forest type, functional traits, and environmental conditions on aboveground biomass (AGB) based on a Gamma generalized linear model (log link). Panel A shows model‐predicted mean AGB for Deciduous, Mixed, and Evergreen forest types; bars represent back‐transformed estimated marginal means from the GLM, and error bars indicate 95% confidence intervals. Panels B–E illustrate partial relationships between AGB and (B) functional diversity, (C) functional dominance, (D) soil fertility (PC1), and (E) climate conditions (PC1), with points showing plot‐level observations, solid lines showing fitted GLM responses, and shaded bands representing 95% confidence intervals.

Functional dominance had the strongest positive effect on AGB (Estimate = 0.787, *t* = 10.62, *p* < 0.001), indicating that forest type dominated by particular traits accumulate substantially more biomass (Figure 2 and Appendix S2). Soil fertility also had a significant positive effect (Estimate = 0.232, t = 3.07, *p* = 0.002), highlighting the importance of nutrient‐rich soils in promoting biomass. Functional diversity and climate conditions did not have significant effects (Estimate = 0.065, t = 1.03, *p* = 0.303 and Estimate = −0.015, t = −0.18, *p* = 0.860), respectively, suggesting limited influence of trait diversity and the specific climatic factors considered (Figure 2 and Appendix S2). Forest type showed significant differences, with mixed forests exhibiting substantially higher AGB than deciduous forests (Estimate = 1.406, t = 7.59, *p* < 0.001) and evergreen forests also showing higher biomass (Estimate = 0.437, t = 3.17, *p* = 0.002) (Figure 2 and Appendix S2). The model fit was strong, with a residual deviance of 72.15 on 193 degrees of freedom compared to a null deviance of 350.54 on 199 degrees of freedom, a dispersion parameter of 0.389, and a low AIC of −303.53, indicating that functional dominance, soil fertility, and forest type are the primary determinants of AGB, while functional diversity and climate have minor or negligible effects in these forest ecosystems (Figure 2 and Appendix S2).

### 3.2 Assessing the Relative Contributions of Functional Traits, Soil, and Climate to Aboveground Biomass (AGB)

**FIGURE 3:**
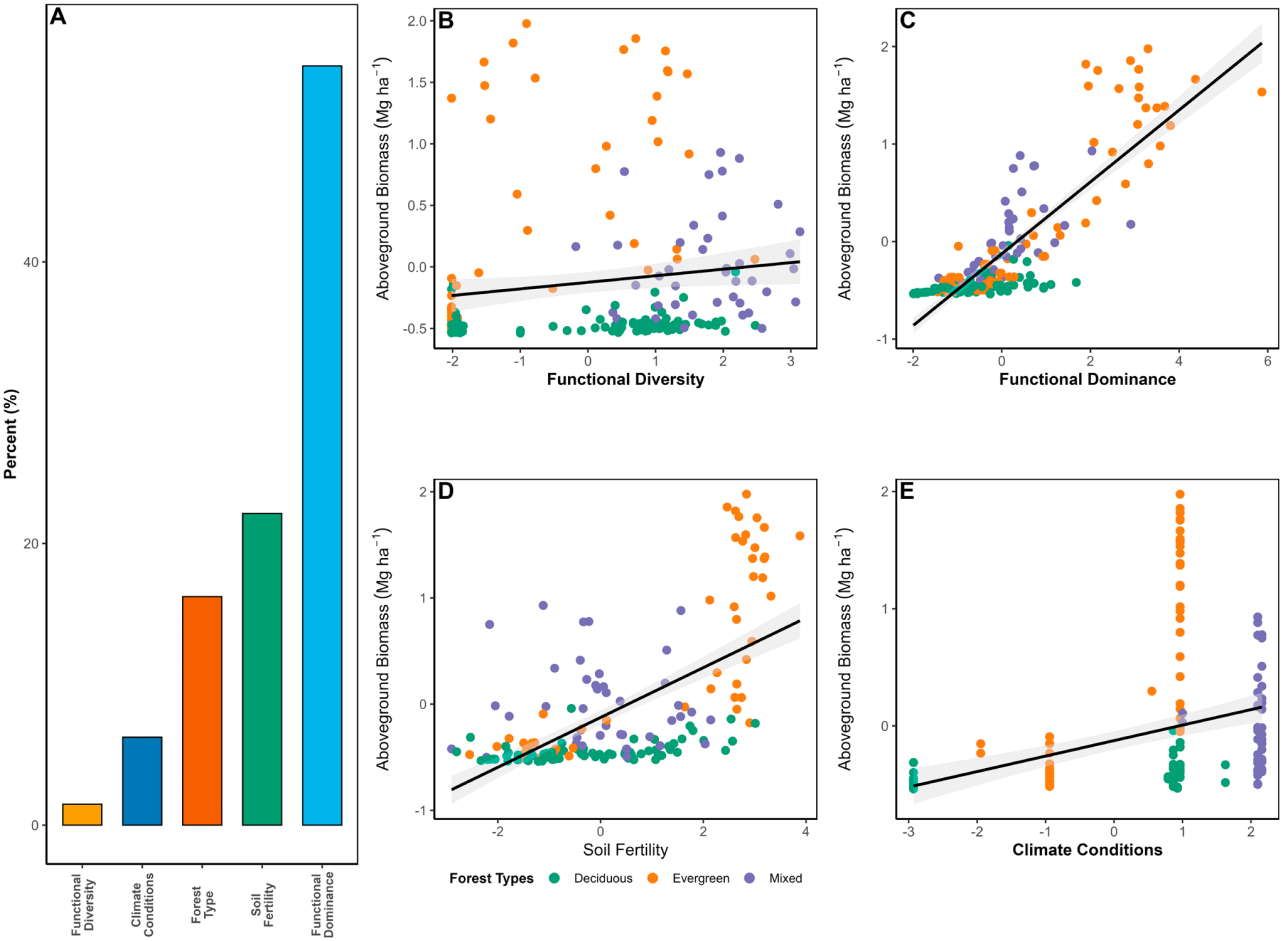
Relative contributions of functional traits, soil fertility, climate, and forest type to aboveground biomass (AGB) and partial relationships between AGB and predictors across forest types. Panel A shows the relative contributions of each predictor to AGB from a multiple linear regression model. Panels B–E show partial relationships between AGB and (B) functional diversity, (C) functional dominance, (D) soil fertility (PC1), and (E) climate conditions (PC1). Points represent plot‐level observations, lines indicate fitted relationships from linear models, and shaded areas denote 95% confidence intervals.

There were significant effects of functional dominance, soil fertility, and climate across forest types. Functional dominance had the strongest positive influence on AGB (Estimate = 0.487, t = 14.92, *p* < 0.001), contributing ~55% to the explained variance, indicating that stands dominated by specific functional traits store more biomass (Figure 3 and Appendix S3). Soil fertility was also positively associated with AGB (Estimate = 0.100, t = 3.00, *p* = 0.003), explaining ~23% of the variance, highlighting the importance of nutrient‐rich soils (Figure 3 and Appendix S3). Climate conditions had a significant negative effect (Estimate = −0.124, t = −3.32, *p* = 0.001), contributing ~6%, suggesting that certain climatic factors may limit biomass accumulation (Figure 3 and Appendix S3). Functional diversity showed a marginal positive effect (Estimate = 0.053, t = 1.91, *p* = 0.0575) with minor contribution (~2%), indicating a weak influence of trait diversity (Figure 3 and Appendix S3).

Forest type differences were partially captured through dummy variables, with Site Mixed exhibiting significantly higher AGB compared to deciduous forests (Estimate = 0.444, t = 5.44, *p* < 0.001), while Site Evergreen did not differ significantly (Estimate = 0.085, t = 1.40, *p* = 0.163), contributing 15%. Overall, functional dominance and soil fertility are the main drivers of aboveground biomass, whereas climate, functional diversity, and forest type explain additional variance, particularly for mixed forests (Figure 3 and Appendix S3).

### 3.3 Ecological and Functional Differentiation Among Forest Types

**FIGURE 4:**
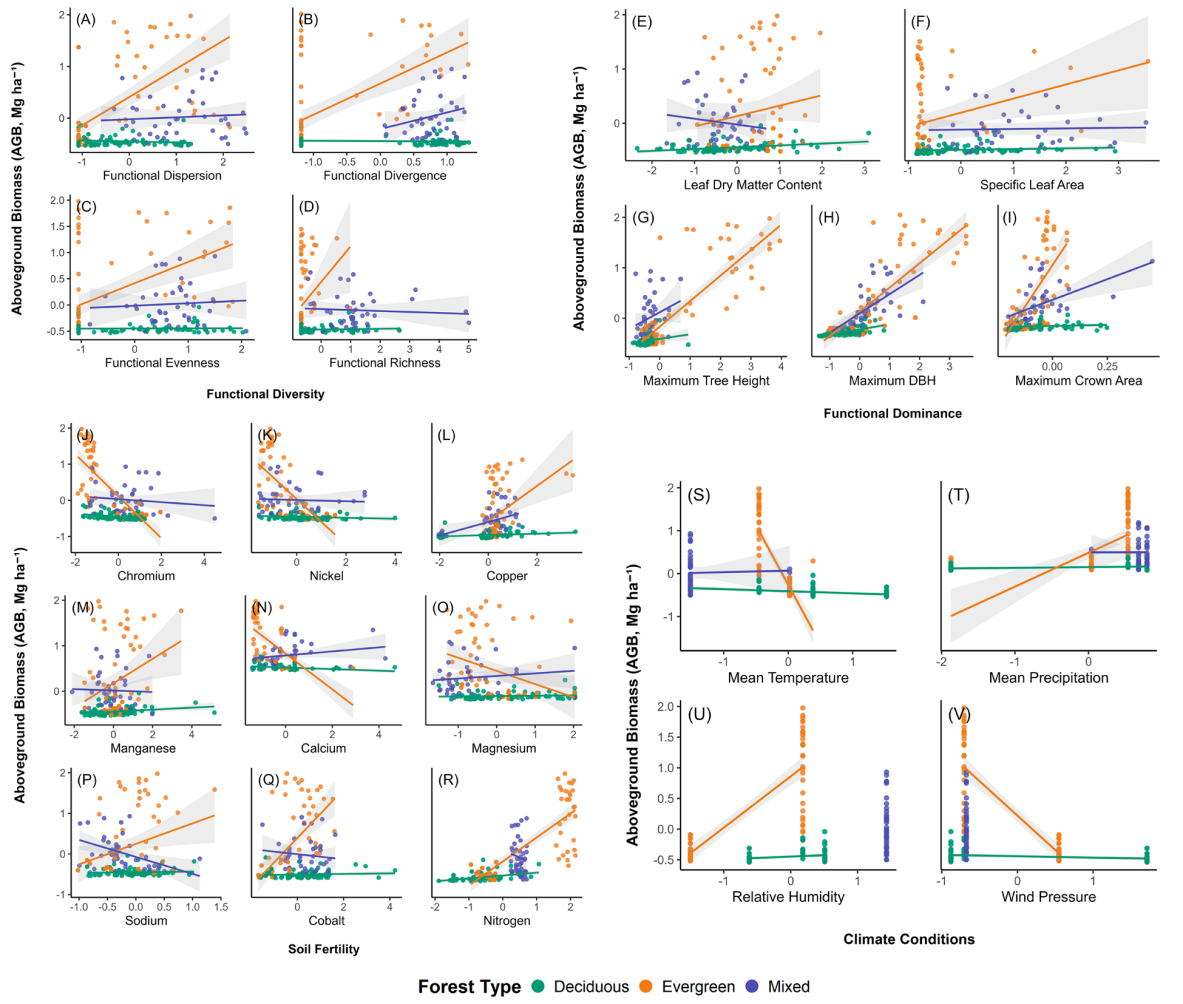
Relationships between abiotic variables and aboveground biomass (AGB) across all forest stand types (Deciduous, Evergreen, and Mixed). Panels (A–D) show functional diversity metrics, panels (E–I) show functional dominance metrics, panels (J–R) show soil fertility variables, and panels (S–V) show climate condition variables. Regression lines represent the relationships between each predictor and AGB for the three forest stand types.

Incorporating all functional trait metrics and environmental variables, functional dominance and soil conditions showed consistently stronger associations with aboveground biomass than functional diversity and climate, with effects varying across forest stand types (Figure 4). Functional metrics varied among forest types, with mixed forests generally exhibiting higher functional diversity and dominance compared to deciduous and evergreen stands, highlighting clear functional and compositional differentiation among forest types (Appendix S5). Species composition also differed clearly among forest types, reflecting variation in abundance, distribution, and successional structure. Deciduous forests were dominated by pioneer species such as *Carissa opaca* Stapf., *Acacia modesta* Wall., and *Mallotus philippensis* Lam., *Celtis australis* auct. indicating disturbance‐adapted communities. Evergreen forests were characterized by late‐successional conifers, including *Pinus gerardiana* Wall., *Pinus wallichiana* A. B. Jackson., *Cedrus deodara* Roxb., *Abies pindrow* Royle, and *Picea smithiana* Wall., whereas mixed forests showed intermediate composition of mid‐ and late‐successional species (Appendix S6).

### 3.4 Variable Importance in Predicting Aboveground Biomass Across Forest Types

**FIGURE 5:**
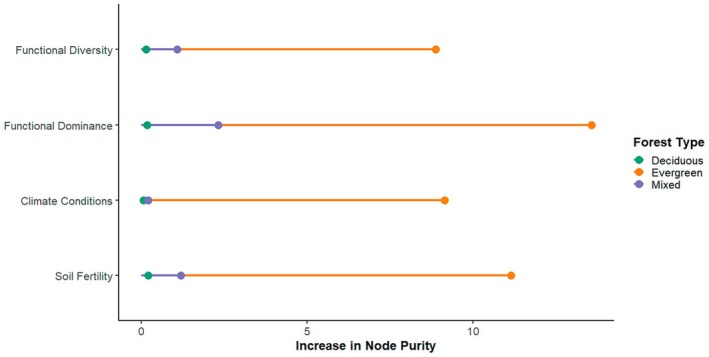
Variable importances for predicting aboveground biomass (AGB) across Deciduous, Evergreen, and Mixed forests using Random Forest, measured by an increase in node purity (IncNodePurity).

Based on Random Forest variable importance measured as increase in node purity. Soil fertility was the most influential predictor of AGB in deciduous forest, accounting for 33.61% of the total relative importance, followed by functional dominance (31.32%) and functional diversity (23.80%), whereas climate conditions contributed the least (11.28%). In mixed forests, functional dominance emerged as the dominant predictor of AGB, explaining 45.67% of the relative importance. Soil fertility (25.06%) and functional diversity (24.47%) showed comparable contributions, while climate conditions had a minimal influence (4.80%), (Figure 5 and Appendix S4). In evergreen forests, functional dominance again showed the highest importance (31.82%), followed closely by soil fertility (28.64%) and climate conditions (20.07%). Functional diversity also contributed substantially (19.47%), indicating a more balanced influence of biotic and abiotic drivers in these forests (Figure 5 and Appendix S4). Overall, functional dominance and soil fertility consistently exerted the strongest influence on AGB across all forest types, whereas the contribution of climate conditions varied markedly, being negligible in mixed forests but relatively more important in evergreen forests. These results highlight forest‐type–specific controls on biomass accumulation and emphasize the dominant role of stand structural and functional attributes in regulating aboveground biomass (Figure 5 and Appendix S4).

## 4 Discussion

Our study provides important insights into the mechanisms shaping aboveground biomass (AGB) in environmentally diverse but resource‐limited forest ecosystems of Pakistan, highlighting the complex interplay between environmental filters, plant functional traits, and forest composition. These findings contribute to the broader understanding of carbon storage dynamics in forest ecosystems, which are vital for biodiversity conservation and climate mitigation globally (Bonan 2008; Mensah et al. 2024; Poorter et al. 2021).

### 4.1 Environmental Filters and Trait‐Mediated Assembly

The differential effects of climate and soil on functional dominance (FDominance) and functional diversity (FD) observed in our study align with community assembly theory (Diamond 1975; MacArthur 1969) and trait‐based ecological frameworks (He et al. 2023; Kraft et al. 2015; Reich 2014; Wang, Hou, et al. 2023; Wang, Song, et al. 2023). Our results show climate acts as a variability‐enhancing environmental filter, promoting both niche differentiation and dominance of resource‐acquisitive traits. This suggests that under favorable climatic conditions, forests can support a wider spectrum of functional strategies, facilitating coexistence through niche complementarity (Tilman et al. 1997; Li and Prentice 2024). Conversely, nutrient‐rich soils appeared to exert a stronger deterministic filter, favoring a subset of competitively superior species characterized by conservative traits, which suppresses trait divergence (Díaz et al. 2004; Zemunik et al. 2015; Mensah et al. 2024). This aligns with evidence from tropical and temperate systems where soil fertility governs species establishment and trait distributions (Clark and Clark 2000; He et al. 2023; Jiang et al. 2023; John et al. 2007).

### 4.2 Functional Traits, Diversity, and Biomass Accumulation

Our finding that functional dominance, rather than trait diversity, is the primary driver of AGB supports the mass ratio hypothesis (Grime 1998), which proposes that ecosystem functioning is disproportionately influenced by the traits of dominant species. This is consistent with studies indicating that traits linked to resource conservation—typical of evergreen species with small, dense leaves—are optimized for biomass accumulation in resource‐limited environments (Pierce et al. 2017; Negreiros et al. 2014; Li and Prentice 2024). Functional diversity, while crucial for supporting species coexistence and resilience (Kunstler et al. 2016; Mouillot et al. 2013; Isbell et al. 2023), did not directly enhance biomass in our system, suggesting that niche complementarity and functional diversity may contribute more to ecosystem stability and resilience than to immediate productivity gains. These results echo findings from tropical secondary forests, where biomass accumulation during succession follows logistic growth patterns influenced by both environmental factors and community functional composition (Chazdon et al. 2007; Chapin et al. 2011; Poorter et al. 2021). Our study extends this understanding by explicitly linking soil nutrient availability and climatic conditions to functional trait distributions and biomass outcomes, demonstrating that soil micro‐ and macro‐nutrients are key drivers of species composition and biomass indirectly by shaping functional traits (Baker et al. 2009; John et al. 2007; Mensah et al. 2024). These trait‐mediated controls on biomass vary systematically across forest successional stages and stand types, reflecting shifts along the resource acquisition–conservation spectrum that underpin changes in community composition and ecosystem functioning.

### 4.3 Forest Composition and Successional Dynamics

These trait‐based differences in functional dominance and diversity are closely linked to successional transitions in forest composition, reflecting a shift from acquisitive to conservative ecological strategies across forest development stages. The significant positive effect of Evergreen forest stands on AGB reinforces the idea that forest structure and dominant functional types act as ecosystem engineers, enhancing carbon storage capacity through greater structural complexity (Chapin et al. 1993; Díaz et al. 2016; Mensah et al. 2024). Mixed forests did not significantly differ from Deciduous forests in biomass, suggesting limited differences in functional composition or transitional successional dynamics under the region's environmental constraints. This aligns with successional theories positing that biomass accumulation is affected both by stand age and changes in species composition and traits over time (Bazzaz 1979; Campetella et al. 2011; Poorter et al. 2021).

Our findings further support these patterns (Appendix S6), showing clear successional partitioning among forest types. Deciduous forests were dominated by pioneer species, such as *Carissa opaca*, *Acacia modesta*, and *Mallotus philippensis* , indicating strong colonization capacity under disturbed and resource‐limited conditions, consistent with early‐successional assembly patterns in resource‐limited ecosystems (Bazzaz 1979; Chazdon et al. 2007; Lebrija‐Trejos et al. 2023). In contrast, evergreen forests were characterized by late‐successional conifers, including *Pinus gerardiana* , *Cedrus deodara* , *Abies pindrow*, and *Picea smithiana* , reflecting mature, structurally stable stands with high carbon storage potential and slow‐growth strategies typical of late‐successional systems (Odum 1969; Reich 2014; Díaz et al. 2016; Li and Prentice 2024). Mixed forests exhibited an intermediate composition with both mid‐ and late‐successional species, indicating transitional successional dynamics and functional overlap between forest types, as commonly observed in successional mosaics and secondary forest systems (Chazdon 2014; Lebrija‐Trejos et al. 2010; Isbell et al. 2023).

The temporal aspect of succession and its interaction with soil properties are crucial considerations. Our results support the notion that stand age and environmental heterogeneity jointly influence functional traits and biomass (Powers et al. 2009; Lebrija‐Trejos et al. 2010). While secondary forests worldwide, which now comprise over half of global forests (Pain et al. 2021), accumulate carbon over time, the maximum biomass and recovery rates are modulated by soil fertility, disturbance history, and community trait composition (Turner 2010; Chazdon et al. 2007; Mensah et al. 2024). This underscores the importance of considering both spatial (edaphic) and temporal (successional) variations in forest management and restoration planning.

### 4.4 Climatic Stress and Biomass Patterns

Contrary to expectations derived from tropical forests (Abbasi et al. 2023), we observed a strong negative direct effect of climate on AGB, likely reflecting the influence of climatic stressors such as drought or temperature extremes in semi‐arid and dry temperate ecosystems. This finding parallels observations from semi‐arid systems where climatic variability limits productivity despite trait optimization (Sankaran et al. 2005; Ferrari et al. 2016; Anderegg et al. 2020; Liu et al. 2023). It highlights the complexity of climate‐biomass relationships and the need to consider both mean climatic conditions and variability or extremes when predicting forest carbon dynamics (Bonan 2008).

### 4.5 Implications for Forest Restoration and Management

Our study reinforces that restoration efforts should focus on promoting species with dominant resource‐conserving traits, particularly Evergreen species, to maximize biomass recovery and carbon sequestration in nutrient‐limited soils (Pywell et al. 2003; Fernandes 2016; Li and Prentice 2024; Mensah et al. 2024). Given the strong indirect effects of functional dominance via forest composition on AGB, managing for trait composition rather than species richness alone could yield better ecosystem functioning outcomes (Mokany et al. 2008; Isbell et al. 2023). This insight is crucial for developing adaptive management strategies tailored to local environmental conditions, especially in regions vulnerable to climatic stress and land degradation such as Pakistan. This is consistent with global syntheses showing that biomass accumulation in forests is primarily driven by functional dominance (mass‐ratio effects) rather than functional diversity, and that these relationships are strongly modulated by climate and soil fertility gradients across biomes (Grime 1998; Díaz et al. 2004; Fotis et al. 2018; Ali et al. 2022; Wang, Hou, et al. 2023; Wang, Song, et al. 2023; Poorter et al. 2021). Integrating our findings with those on tropical and temperate forest carbon dynamics reveals that biomass accumulation is a multi‐faceted process driven primarily by the dominance of resource‐conserving traits, mediated through forest composition and modulated by edaphic and climatic filters. While species diversity and functional diversity enhance ecosystem resilience and niche partitioning, functional dominance exerts a stronger influence on biomass in these systems, supporting the mass ratio theory. Our results call for nuanced forest management approaches that prioritize functional traits and soil fertility to optimize carbon storage and ecosystem sustainability in the face of climate variability and land‐use change.

## 5 Conclusion

This study advances trait‐based ecology by elucidating the complex mechanisms through which aboveground biomass (AGB) in Pakistan's forest ecosystems is regulated by the interplay of functional dominance, forest type, and environmental filters, including climate conditions and soil fertility. Our findings underscore that functional dominance characterized by the prevalence of resource‐conservative traits typically associated with evergreen species is a more potent driver of biomass accumulation than functional diversity. This highlights the primacy of the mass‐ratio hypothesis in shaping ecosystem productivity, particularly in fertile soils that favor the establishment and persistence of competitively superior species. Furthermore, forest type stand composition plays a critical role in regulating carbon storage, with mixed and evergreen‐dominated stands consistently supporting higher AGB than deciduous stands. This pattern demonstrates that stand structure and species dominance strongly mediate ecosystem productivity across forest types in Pakistan.

Environmental filters particularly climatic factors (temperature, precipitation, and humidity) and soil fertility gradients (nutrient availability, e.g., soil nitrogen, and related edaphic conditions), jointly exert strong and sometimes contrasting influences on AGB. While favorable soil fertility enhances biomass accumulation by supporting resource‐efficient and competitively dominant species, climatic variability tends to increase trait diversity and niche differentiation but can also impose constraints on overall biomass production, especially in semi‐arid and dry temperate regions. Our results reinforce the critical importance of integrating plant functional strategies with explicit consideration of tree stand composition, climatic conditions, and soil fertility in forest management and restoration planning. For practical application, forest managers and planners should prioritize the conservation and restoration of evergreen and mixed forest types on fertile soils, as these combinations are most effective in enhancing biomass and carbon sequestration potential. Future research should incorporate temporal dynamics and disturbance regimes to better understand how successional processes and episodic events interact with functional traits and multi component environmental filters (climate and soil fertility) to shape long‐term ecosystem resilience and productivity. Additionally, exploring finer‐scale trait–environment interactions and intraspecific trait variability will provide deeper mechanistic insights and improve predictions of forest responses under changing climate and land‐use scenarios. Ultimately, this trait‐based, multi‐scalar approach offers a robust framework for guiding sustainable forest management and climate mitigation strategies in ecologically and socioeconomically important regions such as Pakistan.

## Author Contributions

**Shahab Ali:** conceptualization (equal), data curation (leading), formal analysis (leading), funding acquisition (equal), investigation (leading), methodology (leading), project administration (equal), software (leading), visualization (equal), writing – original draft (equal), writing – review and editing (equal). **Shujaul Mulk Khan:** funding acquisition (equal), project administration (equal), supervision (equal), writing – original draft (equal). **Henrik Balslev:** supervision (equal), validation (equal), visualization (equal), writing – original draft (equal), writing – review and editing (equal). **David P. Edwards:** conceptualization (equal), supervision (equal), validation (equal), visualization (equal), writing – original draft (equal), writing – review and editing (equal).

## Funding

The authors have nothing to report.

## Conflicts of Interest

The authors declare no conflicts of interest.

## Supporting information


**Appendix S1:** Forest types, location, coordinate elevation, slope angle, area, mean temperature, and mean precipitation of the study area.
**Appendix S2:** Summary of the Gamma generalized linear model (log link) assessing the effects of functional dominance, functional diversity, soil fertility, climate, and forest type on aboveground biomass (AGB).
**Appendix S3:** Parameter estimates, standard errors, test statistics, *p*‐values, and relative importance of predictors from the Gamma generalized linear model (log link) explaining variation in aboveground biomass (AGB) across forest types.
**Appendix S4:** Site‐specific variable importance derived from the Random Forest model, showing mean increase in node purity and relative importance (%) of climate conditions, functional diversity, functional dominance, and soil fertility in explaining aboveground biomass across Deciduous, Mixed, and Evergreen forest types.
**Appendix S5:** Mean values ± standard error (SE) of functional diversity, functional dominance, soil fertility, climate conditions, and aboveground biomass (AGB) in Deciduous, Evergreen, and Mixed forest stands.
**Appendix S6:** Dominant species composition, abundance, relative abundance (%), frequency (%), and successional status of the major tree and shrub species across Deciduous, Evergreen, and Mixed forest stand types.

## Data Availability

The data supporting the findings of this study are openly available in Zenodo at https://zenodo.org/records/19609795 (DOI: 10.5281/zenodo.19609795).
