# Supplementary material for: Plant‐Trait Syndromes and Environmental Filtering Drive Biomass Ecology in Resource‐Limited Forest Ecosystems
Source: Ecol Evol. 2026 Jul 7;16(7):e73949. doi: 10.1002/ece3.73949 (PMC13341152; doi:10.1002/ece3.73949)
Supplement: Supplementary file 1 — Appendix S1: Forest types, location, coordinate elevation, slope angle, area, mean temperature, and mean precipitation of the study area. Appendix S2: Summary of the Gamma generalized linear model (log link) assessing the effects of functional dominance, functional diversity, soil fertility, climate, and forest type on aboveground biomass (AGB). Appendix S3: Parameter estimates, standard errors, test statistics, p‐values, and relative importance of predictors from the Gamma generalized linear model (log link) explaining variation in aboveground biomass (AGB) across forest types. Appendix S4: Site‐specific variable importance derived from the Random Forest model, showing mean increase in node purity and relative importance (%) of climate conditions, functional diversity, functional dominance, and soil fertility in explaining aboveground biomass across Deciduous, Mixed, and Evergreen forest types. Appendix S5: Mean values ± standard error (SE) of functional diversity, functional dominance, soil fertility, climate conditions, and aboveground biomass (AGB) in Deciduous, Evergreen, and Mixed forest stands. Appendix S6: Dominant species composition, abundance, relative abundance (%), frequency (%), and successional status of the major tree and shrub species across Deciduous, Evergreen, and Mixed forest stand types. [file ECE3-16-e73949-s001.docx]

**Appendix S1** Forest types, location, coordinate elevation, slope angle, area, mean temperature, and mean precipitation of the study area.

| **Forest types** | **Location** | **Coordinates** | **Elevation m.a.s.l** | **Slope Angle (^°^) Average** | **Area Km^2^** | **Mean Annual Temperature (◦C)** | **Mean Annual Precipitation (mm)** | **References** |
| --- | --- | --- | --- | --- | --- | --- | --- | --- |
| i.Sub-tropical thorn Forest  Kirthar National Park (Deciduous ). | Sindh | 67°10' E to 67°55' E longitudes to 25°13' N to 26°12' N latitude | 56–302 | Flat | 3087.33 | 33.8 | 245.3 | Ali et al., 2023b |
| ii.Sub-tropical broad-leaved forest, Margalla Hills National Park (Deciduous). | Islamabad | 33°0400 N to 33°0550 N longitude to 73°0200 E to 73°0440 E latitude | 555–1117 | 21.7 | 203 | 27.8 | 1572.1 | Ali et al., 2023b |
| iv.Moist temperate mix forest, Murree Forest Division (Mixed). | Punjab | 33°520 N to 33°590 N and 73°240 E to 73°310 E | 1249–2892 | 29.55 | 200 | 17.8 | 1596.1 | Ali et al., 2023b |
| iii.Dry temperate conifer forest, Kumrat Vallay, Dir Upper, KPK (Evergreen). | KPK | 35°00 N to 28°00 N to 72°00 E to 20°00 E longitude | 1040–2566 | 22 | 870 | 23.4 | 1371.8 | Ali et al., 2023b |
| v.Dry temperate pure *Pinus gerardiana* forest, Koh e Suleiman mountain Range ( Evergreen). | Balochistan | 31°00 N to 36°00 N latitude and 69°00 E to 59°00 E | 1841–2282 | 21.85 | 260 | 25.9 | 299.0 | Ali et al., 2023b |

**Appendix S2** Summary of the Gamma generalized linear model (log link) assessing the effects of functional dominance, functional diversity, soil fertility, climate, and forest type on aboveground biomass (AGB).

| Predictor | Estimate | Std. Error | t value | P value |
| --- | --- | --- | --- | --- |
| (Intercept) | -1.99180 | 0.07887 | -25.253 | < 2e-16 |
| FD_PC1 | 0.06535 | 0.06324 | 1.033 | 0.30271 |
| FDom_PC1 | 0.78725 | 0.07416 | 10.615 | < 2e-16 |
| Soil_PC1 | 0.23181 | 0.07554 | 3.069 | 0.00246 |
| Climate_PC1 | -0.01493 | 0.08477 | -0.176 | 0.86041 |
| SiteMixed | 1.40644 | 0.18539 | 7.586 | 1.36e-12 |
| SiteEvergreen | 0.43705 | 0.13790 | 3.169 | 0.00178 |

## Appendix S3 Parameter estimates, standard errors, test statistics, p-values, and relative importance of predictors from the Gamma generalized linear model (log link) explaining variation in aboveground biomass (AGB) across forest types.

| Predictor | Estimate | Std. Error | t value | P value | Relative Importance (%) |
| --- | --- | --- | --- | --- | --- |
| (Intercept) | -0.244 | 0.035 | -7.03 | <0.001 | NA |
| FD_PC1 | 0.053 | 0.028 | 1.91 | 0.0575 | 1.82 |
| FDom_PC1 | 0.487 | 0.033 | 14.92 | <0.001 | 55.68 |
| Soil_PC1 | 0.100 | 0.033 | 3.00 | 0.0031 | 22.16 |
| Climate_PC1 | -0.124 | 0.037 | -3.32 | 0.0011 | 5.81 |
| SiteMixed | 0.444 | 0.082 | 5.44 | <0.001 | NA |
| SiteEvergreen | 0.085 | 0.061 | 1.40 | 0.163 | NA |

## Appendix S4 Site-specific variable importance derived from the Random Forest model, showing mean increase in node purity and relative importance (%) of climate conditions, functional diversity, functional dominance, and soil fertility in explaining aboveground biomass across Deciduous, Mixed, and Evergreen forest types.

| Site | Variable | Mean Increase in Node Purity | Relative Importance (%) |
| --- | --- | --- | --- |
| Deciduous | Climate Conditions | 0.066015007 | 11.28 |
| Deciduous | Functional Diversity | 0.139317997 | 23.8 |
| Deciduous | Functional Dominance | 0.183350775 | 31.32 |
| Deciduous | Soil Fertility | 0.196762076 | 33.61 |
| Mixed | Climate Conditions | 0.225779754 | 4.8 |
| Mixed | Functional Diversity | 1.150111336 | 24.47 |
| Mixed | Functional Dominance | 2.146906254 | 45.67 |
| Mixed | Soil Fertility | 1.177802185 | 25.06 |
| Evergreen | Climate Conditions | 8.489604171 | 20.07 |
| Evergreen | Functional Diversity | 8.237348451 | 19.47 |
| Evergreen | Functional Dominance | 13.46038289 | 31.82 |
| Evergreen | Soil Fertility | 12.11478252 | 28.64 |

**Appendix S5** Mean values ± standard error (SE) of functional diversity, functional dominance, soil fertility, climate conditions, and aboveground biomass (AGB) in Deciduous, Evergreen, and Mixed forest stands.

| Forest Type | Functional Diversity | Functional Dominance | Soil Fertility | Climate | AGB |
| --- | --- | --- | --- | --- | --- |
| Deciduous | 0.023 ± 0.073 | -0.250 ± 0.039 | -0.020 ± 0.033 | 0.143 ± 0.011 | -0.449 ± 0.009 |
| Evergreen | -0.548 ± 0.077 | 0.154 ± 0.080 | -0.020 ± 0.028 | -0.185 ± 0.013 | 0.208 ± 0.100 |
| Mixed | 0.907 ± 0.069 | 0.241 ± 0.046 | 0.080 ± 0.049 | 0.003 ± 0.008 | 0.018 ± 0.063 |

**Appendix S6** Dominant species composition, abundance, relative abundance (%), frequency (%), and successional status of the major tree and shrub species across Deciduous, Evergreen, and Mixed forest stand types

| Forest_Type | Dominant_Species | Abundance | Relative_Abundance | Frequency_Percent | Successional_Status |
| --- | --- | --- | --- | --- | --- |
| Deciduous | *Carissa opaca* Stapf. | 1426 | 22.38 | 45.68 | Pioneer |
| Deciduous | *Acacia modesta* Wall. | 550 | 8.63 | 69.14 | Pioneer |
| Deciduous | *Mallotus philippensis* Lam. | 344 | 5.4 | 34.57 | Mid |
| Deciduous | *Celtis australis* auct. | 247 | 3.88 | 30.86 | Mid |
| Deciduous | *Prosopis juliflora* Sw. DC. | 179 | 2.81 | 19.75 | Pioneer |
| Evergreen | *Pinus gerardiana* Wall. | 1594 | 44.17 | 47.5 | Late |
| Evergreen | *Pinus wallichiana* A. B. Jackson. | 333 | 9.23 | 23.75 | Late |
| Evergreen | *Cedrus deodara* Roxb. | 266 | 7.37 | 15 | Late |
| Evergreen | *Abies pindrow* Royle. | 201 | 5.57 | 20 | Late |
| Evergreen | *Picea smithiana* Wall. | 95 | 2.63 | 12.5 | Late |
| Mixed | *Viburnum grandiflorum* Wall. | 685 | 13.01 | 45 | Mid |
| Mixed | *Pinus wallichiana* A. B. Jackson. | 654 | 12.42 | 72.5 | Late |
| Mixed | *Diospyros virginiana* L. | 214 | 4.07 | 25 | Mid |
| Mixed | *Castanea dentate* Marshall. | 208 | 3.95 | 22.5 | Mid |
| Mixed | *Aesculus indica* Wall. | 204 | 3.88 | 32.5 | Mid |
| Mixed | *Abies pindrow* Royle. | 149 | 2.83 | 25 | Late |
